# Representativeness of microsatellite distributions in genomes, as revealed by 454 GS-FLX Titanium pyrosequencing

**DOI:** 10.1186/1471-2164-11-560

**Published:** 2010-10-12

**Authors:** Jean-Francois Martin, Nicolas Pech, Emese Meglécz, Stéphanie Ferreira, Caroline Costedoat, Vincent Dubut, Thibaut Malausa, André Gilles

**Affiliations:** 1 Centre de biologie et de gestion des Populations (Montpellier SupAgro, INRA, IRD, CIRAD), Campus International de Baillarguet, CS30016, Montferrier sur Lez cedex, France; 2 Aix-Marseille Université, CNRS, IRD, UMR 6116 - IMEP, Equipe Evolution Génome Environnement, Centre Saint-Charles, Case 36, 3 place Victor Hugo, 13331 Marseille Cedex 3, France; 3 Genoscreen, Genomic Platform and R&D, Campus de l'Institut Pasteur, 1 rue du Professeur Calmette, Bâtiment Guérin, 4ème étage, 59000 Lille, France; 4 Institut National de la Recherche Agronomique, UMR 1301, Equipe BPI, 400 route des Chappes, BP 167, 06903 Sophia-Antipolis Cedex, France

## Abstract

**Background:**

Microsatellites are markers of choice in population genetics and genomics, as they provide useful insight into patterns and processes as diverse as genome evolutionary dynamics and demographic processes. The acquisition of microsatellites through multiplex-enriched libraries and 454 GS-FLX Titanium pyrosequencing is a promising new tool for the isolation of new markers in unknown genomes. This approach can also be used to evaluate the extent to which microsatellite-enriched libraries are representative of the genome from which they were isolated. In this study, we deciphered potential discrepancies in microsatellite content recovery for two reference genomes (*Apis mellifera *and *Danio rerio*), selected on the basis of their extreme heterogeneity in terms of the proportions and distributions of microsatellites on chromosomes.

**Results:**

The *A. mellifera *genome, in particular, was found to be highly heterogeneous, due to extremely high rates of recombination, with hotspots, but the only bias consistently introduced into pyrosequenced multiplex-enriched libraries concerned sequence length, with the overrepresentation of sequences 160 to 320 bp in length. Other deviations from expected proportions or distributions of motifs on chromosomes were observed, but the significance and intensity of these deviations was mostly limited. Furthermore, no consistent adverse competition between multiplexed probes was observed during the motif enrichment phase.

**Conclusions:**

This approach therefore appears to be a promising strategy for improving the development of microsatellites, as it introduces no major bias in terms of the proportions and distribution of microsatellites.

## Background

Several fields of research in biology and genetics make use of molecular markers to address various questions relating to population biology, quantitative genetics, forensics, parentage analyses and conservation genetics, for example [1]. In this context, microsatellites, or simple sequence repeats (SSRs) are commonly used, as they have a number of desirable features, such as ease of use, codominance and high mutation rates [2]. In population genetics and genomics, the polymorphism of microsatellite markers is thought to provide insight into the evolutionary events occurring within and between populations, in individual genomes [3]. The markers used are generally assumed to be representative in population genetics. However, this assumption of representativeness is particularly critical in several other fields, such as population genomics [4] and genome mapping [5,6], because such studies explicitly require large numbers of markers representative of the evolution of the entire genome.

Information about the representativeness of markers is directly available for sequenced genomes. By contrast, the extent to which microsatellite loci may be considered independent, and the extent to which their relative proportions and distributions may be considered representative in unsequenced genomes remain largely unknown. This is largely because only small numbers of sequences tend to be available for such genomes, as they are generally obtained by strategies based on Sanger sequencing, which is very time-consuming. The advent of new high-throughput sequencing technologies has made it possible to produce extensive sequence data for previously unsequenced genomes, providing new opportunities to assess the extent to which microsatellite sequences are representative. In particular, the coupling of multiple, simultaneous enrichments of microsatellite motif-enriched libraries (hereafter referred to as multiplex enrichments) with high-throughput 454 pyrosequencing provides a theoretically efficient procedure for efficient microsatellite isolation. However, the question of the extent to which the sequences obtained in this way are representative of the genome sampled has never been addressed. In this study, we aimed to determine whether multiplex enrichment followed by pyrosequencing introduced any bias, in terms of i) the relative proportions of microsatellite motifs and ii) their distribution on and between the chromosomes of the organisms studied.

Heterogeneity in the proportions and distributions of microsatellite may arise for several reasons, related to the combination of various evolutionary processes [7]. Microsatellites are clearly not evenly distributed within genomes, because their frequencies in coding and non-coding sequences are different [8]. Moreover, comparisons of frequency data for different genomes (even for closely related species) strongly suggest that microsatellite distribution is not merely a reflection of the base composition of the genome, with the DNA repair system instead playing an important role in determining the microsatellite distributions of different species [9-11]. Variability in the evolutionary dynamics of microsatellite loci has been demonstrated in eukaryotes [10,12-15]. Recombination rate plays an even more important role. Recombination maintains diversity against purifying selection involving both linkage disequilibrium and homogenization, but this process also reorganizes the diversity of bivalent chromosomes. Recombination strongly influences both the polymorphism and distribution of microsatellite loci [16,17]. Finally, genome duplication is recognized as one of the most important driving forces in genome evolution [18-20]. Evolutionary events of this type create paralogous sequences that may later be reshuffled in the diploidization process [21], greatly modifying the structure of the genome [22,23] and therefore also the distribution of microsatellite sequences [21]. This diversity of mechanisms and their combination produce different proportions and distributions of microsatellite motifs within the genome that may be unique to the genome concerned.

We addressed the key question relating to whether the pyrosequencing of multiplex-enhanced libraries raises any representativeness issues of general concern.

We decided to tackle this question by studying the complete published genomes of model organisms with highly heterogeneous genomes, potentially resulting in distortion of the relative proportions of microsatellite motifs and increasing the extent to which certain areas of the genome are likely to be under- or overrepresented when pyrosequencing multiplex-enriched libraries. The honey bee, *Apis mellifera *Linnaeus, 1758 [Insecta: Hymenoptera: Apidae], is a good candidate organism for studies of heterogeneous microsatellite distributions within the genome. This species has 16 chromosomes for a total genome size of 262 Mb and has a particularly high recombination rate (20 times higher than that of humans, for example [17,24]). However, despite the relative uniformity of the rate of recombination over a large scale, the genome is nonetheless punctuated by numerous recombination hotspots. This feature is of particular interest in our framework, as at least some SSRs are considered to act as recombination hotspots [25,26]. The observed positive correlation between the occurrence of microsatellite loci and recombination rate shapes the distribution of microsatellites within the honey bee genome, with these markers tended to be found within recombination hotspots [16,17], resulting in a highly heterogeneous distribution of microsatellites on the chromosomes (see Additional file 1, Additional file 2, Figure S1, Additional file 3, Figure S2 and Additional file 4, Figure S3).

The zebrafish, *Danio rerio *(Hamilton, 1822) [Actinopterygii: Cypriniformes: Cyprinidae], was also selected as a model organism for this study. It has an estimated haploid size of 1.5 Gb and the telomeric and centromeric chromosome regions have been shown to be rich in long arrays of various mono-, di-, tri-, tetra-and hexanucleotide motifs in telesostean fishes [27]. However, it remains difficult to assess the representativeness of microsatellites in telomeric and centromeric regions with traditional isolation techniques [28]. An analysis of the distribution of microsatellites in the published genome sequence of the zebrafish, as detailed in the Additional file 1, provided information consistent with published findings, with a heterogeneous distribution of microsatellite loci such that these loci tend to concentrated in the telomeric regions.

*In silico *analyses of the assembled genome sequences of the two model organisms studied showed that the different dynamics of genome evolution in these two species translated into differences in the heterogeneity of microsatellite distribution, through different mechanisms. These published sequences provide a useful framework for assessing the representativeness of the microsatellite sequences obtained by the pyrosequencing of multiplex-enriched libraries. For comparisons between the *in silico *reference sequences and pyrosequencing data, we generated enriched libraries based on the hybridization of biotinylated probes -- a procedure widely used in microsatellite development [29] -- multiplexing various sets of hybridization probes selected after a comparative analyses of the many metazoan genomes available (see Methods and Additional file 1, Table S1 for details). We then constructed libraries from these enriched subsets of total DNA and sequenced them with the 454 Titanium pyrosequencing reagents (Roche Diagnostics). We assessed the extent to which enriched libraries were representative by comparing simulated and observed indicators: distribution on the chromosomes, proportion and abundance of microsatellite motifs and the length of the sequences obtained.

## Results

### Isolation and pyrosequencing of microsatellite loci

Three libraries enriched with two, five and eight probes were constructed for *A. mellifera *and *D. rerio*. The sequences obtained for each library are provided in Additional file 5. The number of sequences obtained is summarized in Table 1 and was smaller than expected on the basis of the manufacturer's specifications (20,000 to 50,000 sequences for 1/16th of a Titanium series XLR70 PicoTiter plate) for all libraries, particularly for *D. rerio*. The underlying cause of this discrepancy may have been specific to this organism or the number of biotinylated beads may occasionally have been misestimated during preparation of the 454 library. Sequencing artifacts would be observed for both species, whereas problems associated with one of the three enrichment protocols (two-probe, five-probe and eight-probe enrichment) would be reflected in the corresponding libraries, regardless of the organism considered.

**Table 1:** Summarized data for sequences from multiplex-enriched libraries, for both *A. mellifera *and *D. rerio*

	Total no. of sequences	Length > 80 bp	Containing microsatellites	Total number of sequences obtained with primer	Unique sequences	Consensus sequences
*D. rerio *2 probes	14789	9813	6758	283	238	45
*D. rerio *5 probes	12789	8783	7096	382	346	36
*D. rerio *8 probes	15833	10733	9123	333	301	32
						
*A. mellifera *2 probes	22940	14706	11217	138	103	35
*A. mellifera *5 probes	22189	14706	10958	166	139	27
*A. mellifera *8 probes	27392	20152	16283	237	176	61

		**Length > 80 bp/total**	**Containing microsatellites**	**Percentage of sequences obtained with primer**		
		
*D. rerio *2 probes		66.4%	68.9%	4.2%		
*D. rerio *5 probes		68.7%	80.8%	5.4%		
*D. rerio *8 probes		67.8%	85.0%	3.7%		
						
*A. mellifera *2 probes		64.1%	76.3%	1.2%		
*A. mellifera *5 probes		66.3%	74.5%	1.5%		
*A. mellifera *8 probes		73.6%	80.8%	1.5%		

For all libraries, 65% to 76% of the sequences obtained were within the size range selected for further analysis (i.e. more than 80 bp long). The percentage of these sequences with a microsatellite motif was between 69% and 85%, depending to the species and the probe set used (Table 1). Enrichment was less efficient for *A. mellifera *than for *D. rerio *(χ^2 ^= 1,106.39, Df = 9, P < 0.05). Motif enrichment greatly increased the total number of sequences containing microsatellites over that obtained with a protocol not including enrichment (maximum of 7.72% in other comparable studies [30-33]). Above all, motif enrichment provided many more reads for these loci, making it possible to detect the polymorphism in flanking regions of particular interest for primer design [34-36]. The bioinformatics pipeline QDD [37] was used to filter for redundancy and to check for multiple copies, resulting in a final set of sequences for which we were able to design primers. The higher redundancy observed in *A. mellifera *is not surprising, because the genome of this species is only one seventh the size of that of *D. rerio*, so random high-throughput sampling of the enriched libraries is more rapidly saturated. This feature may have affected the number of sequences usable for primer design, but the most striking difference between *A. mellifera *and *D. rerio *was the high proportion (60% to 64%) of sequences in *A. mellifera *discarded by filtering. This filtering was based on the selection of a percentage of similarity for use as a threshold for homology (here, 95%). For *D. rerio*, only 20% to 25% of the sequences were discarded. This difference may reflect a signature of transposition, recombination and/or duplication events in *A. mellifera *[24]. This procedure for sequence filtering opens up new possibilities for the optimization of primer design. Single copy-specific primers minimize the number of null alleles and multiple PCR products [38]. Finally, for the two-, five- and eight-probes libraries, we obtained between 138/166/237 loci in *A. mellifera *and 283/382/333 loci in *D. rerio *for which primers were successfully designed (in most cases, multiple pairs of primers were used for each single locus). We deliberately chose to use stringent parameters for primer design (see Methods for details), as we considered efficiency to be more important in time-consuming laboratory work (the testing of primers sets), but other parameter values in the quantity/quality trade-off for primers would be as valid as those used here, and could easily be implemented in the QDD pipeline. However, we used this set of parameters in the application of this methodology to the isolation of microsatellites as it led to the rapid and efficient development of 105 microsatellite loci in *Zingel asper *(Linnaeus, 1758) [Actinopterygii: Perciformes: Percidae] (see Additional file 1, Table S2).

### Representativeness of the genome features as revealed by 454 pyrosequencing

The analysis *in silico *of the reference genomes digested with *Rsa*I is detailed in Additional file 2, Figure S1. However, it is useful to bear in mind the general tendencies of the reference genome. The *A. mellifera *and *D. rerio *genomes are not structured in the same way. The *A. mellifera *genome has one microsatellite motif (AG) overrepresented with respect to theoretical homogeneous relative proportions, and *D. rerio *has two such motifs (AC and AG motifs). The chromosomes of *A. mellifera *have many stochastic high densities of microsatellites and an unbalanced distribution of loci in one arm for most chromosomes (Additional file 4, Figure S3). By contrast, the *D. rerio *genome, after digestion with *Rsa*I, was found to contain an excess of microsatellites in the telomeric regions of the chromosomes (Additional file 4, Figure S3).

**Figure 1:**
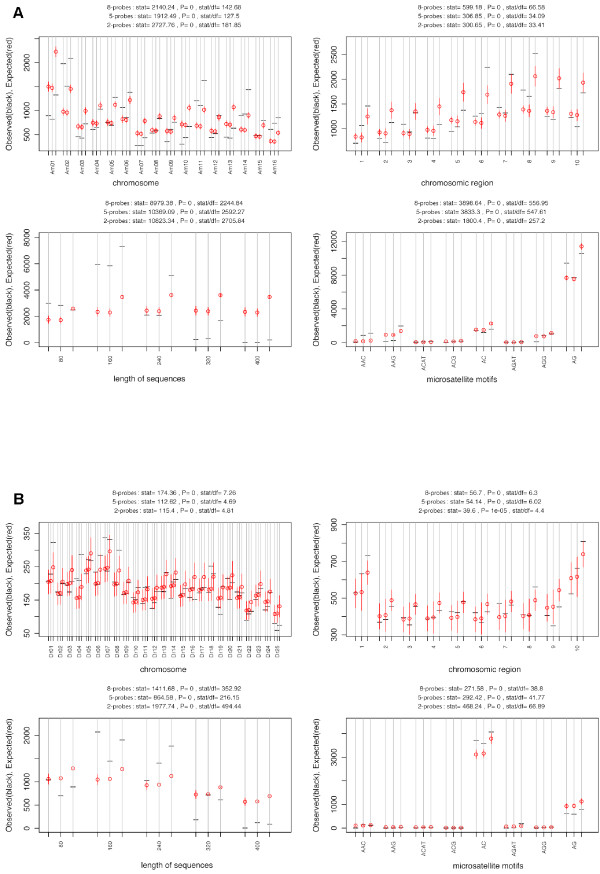
**Comparison of 454 microsatellite sequence data and the reference genome for *Apis mellifera *(A) and *Danio rerio *(B), for each of the one-way tables considered: chromosome, chromosome region, length of sequences and microsatellite motifs**. For each table, the 95% confidence interval of the expected number of microsatellites based on the published *Rsa*I-digested genome distribution is shown in red whereas the observed number (obtained by 454 pyrosequencing) is shown in black. For each one-way table, the 2- 5- and 8-probe analyses are shown.

We assessed the representativeness of the sequences obtained from the multiplex-enriched libraries, using the distribution features of enriched fragments from the eight-probe multiplex-enriched libraries for both *A. mellifera *and *D. rerio*. We evaluated the extent to which these distributions matched the corresponding assembled reference genomes, to detect deviations in the enriched libraries. The variables used in this analysis were: *i*, the different chromosomes; *j*, the regions of each chromosome; *k*, the length of the sequences obtained and *l*, the motifs (see Additional file 1 for details and definitions). Reference distributions of these parameters were obtained by *in silico *digestion of the two published reference genomes with *Rsa*I (Figure 1). The results obtained for each of the above variables in the eight-probe context are described below.

#### Location on the various chromosomes

An analysis of the location of the motifs on the various chromosomes indicates whether certain chromosomes are favored over other in enrichment and pyrosequencing methods. For *D. rerio*, we observed no significant deviation from the theoretical expected distribution of microsatellites for 20 of the 25 chromosomes (80%). Three of the five chromosomes displaying significant discrepancies (#1, 4 and 8) were overrepresented with respect to the number of microsatellites found in the 454 sequences, whereas the other two (#16 and 25) were underrepresented (Figure 1). The situation was very different for *A. mellifera*, for which no significant bias in the experimental results was observed for only four of the 16 chromosomes (25%). For the 12 chromosomes for which a significant difference from the expected distribution was observed, seven (#2, 5, 6, 11, 14, 15 and 16) were overrepresented and five (#1, 3, 7, 10 and 13) were underrepresented (Figure 1).

#### The regions of each chromosome

The two genomic reference models (obtained by digesting the chromosomes with *Rsa*I) displayed different distributions of microsatellites between the 10 regions of each chromosome, with each region defined as 10% of the chromosome length (for more details see Additional file 2, Figure S1). In *D. rerio*, the frequency of microsatellites was much higher in the telomeric regions of the chromosomes than elsewhere, whereas, in *A. mellifera*, microsatellite frequency was generally highest in region 8, but with differences between the chromosomes (see Additional file 2, Figure S1 for details). The 454 data distribution fit the reference chromosome regions better for *D. rerio*, for which seven regions (70%) were correctly represented (Figure 1). The other three regions were regions 1 and 8, which were overrepresented, and region 9, which was underrepresented. For *A. mellifera*, four regions (40%) were correctly represented by the experimental data (Figure 1), three were overrepresented (regions 1, 6 and 8) and three were underrepresented (regions 2, 4, and 5).

#### Sequence length distribution

**Figure 2:**
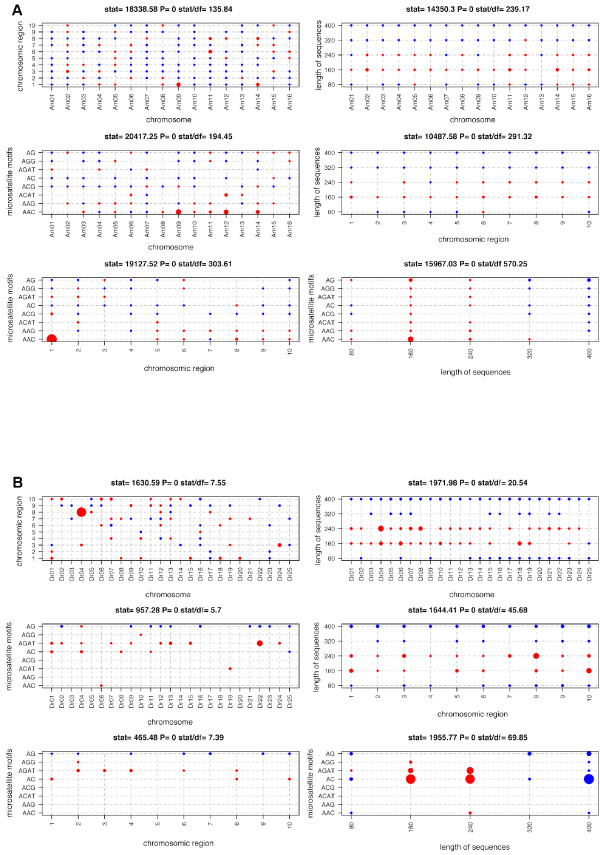
**Comparison of 454 microsatellite sequence data (for the 8 probes) for *Apis mellifera *(A) and *Danio rerio *(Bb), for each of the six two-way tables considered: chromosome × chromosome region, chromosome × length of sequences, chromosome × microsatellite motifs, chromosome region × length of sequences, chromosome region × microsatellite motifs and length of sequences × microsatellite motifs**. "Stat" refers to the overall χ^2 ^statistics for the test of independence between the two variables defining each table. For each cell of the table, a red dot corresponds to an observed number significantly higher than expected under the hypothesis of independence between the two variables defining the table, a blue dot corresponds to an observed number significantly lower than expected under the hypothesis of independence between the two variables defining the table; no dot indicates a non-significant deviation. The size of the dot is inversely proportional to the P-value, with scaling such that comparisons of sizes between tables are meaningful.

The distribution of sequences lengths deviated significantly from the expected reference proportions for both the biological models studied (Figure 1 and Figure 2), with overrepresentation of sequences between 161 and 320 bp in size (up to three times as many such sequences as expected) and an underrepresentation of other lengths (80-160 and 321-480). This pattern probably resulted from a problem with the 454 pyrosequencing process for this run.

#### Relative abundance of the eight selected microsatellite motifs

The reference relative proportions of motifs were generally correctly estimated from 454 data for *D. rerio*. However, marginal significant deviations were observed for the AC motif, which was overrepresented, and the AG motif, which was underrepresented (Figure 1). For *A. mellifera*, the relative proportions of only four of the eight selected motifs were correctly estimated (Figure 1). Two motifs (AAG and AAC) were overrepresented and the two dinucleotide motifs (AG and AC) were underrepresented.

Thus, the results obtained for *D. rerio *were more representative than those for *A. mellifera *(Figure 2). Sequence length distribution displayed a similar bias in the two species, and the other features identified highlighted only occasional deviations related to the organism studied, with no other bias linked to the methodology itself detected.

### Is the multiplexing of probes a suitable strategy and are there biases into the enriched libraries obtained?

Multiplexing, with the simultaneous use of up to eight probes in the enrichment process, overcomes the problems of probe selection in the absence of knowledge about the genome. However, competition between probes is a potential problem that may arise with this approach. The nested design used here for the two-, five- and eight-probe enriched libraries made it possible to test whether the addition of probes led to particular deviations in motif distribution.

No adverse effects of competition were consistently detected in the motif-enriched libraries. Multiplexing strategies based on eight motifs may therefore be a useful surrogate method for the evaluation of microsatellite diversity in unknown genomes. The deviations in the proportions of the AC and AG motifs were of only marginal significance and differed between the enriched libraries, varying with the number of probes: from over- to underrepresentation with increasing number of probes for AG and from non-significant to underrepresentation for AC. Nevertheless, these shifts were observed only in *A. mellifera*, and may be accounted for by the highly heterogeneous nature of the genome of this species, as repeatedly demonstrated in previous studies, and by the higher frequency of these motifs than of the others studied, increasing the sensitivity of detection for statistically significant deviations.

**Figure 3:**
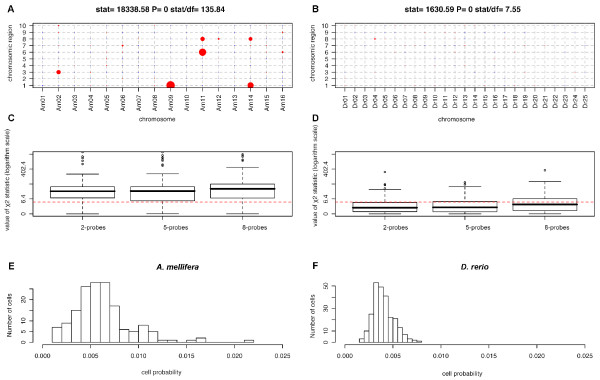
**χ^2 ^test of independence for the two-way table chromosome × chromosome region, for *Apis mellifera *(A) and *Danio rerio *(B)**. The size of the dot is inversely proportional to the P-value, with scaling such that comparisons of sizes between tables are meaningful. Boxplots of the χ^2 ^statistics for each of two, five and eight probes are indicated on the X-axis. A logarithmic scale is used on the Y-axis for the χ^2 ^values. The red dotted line represents the significance limit for *Apis mellifera *(C) and *Danio rerio *(D). The distribution of the cell probabilities from the chromosome × chromosome region two-way table (figure 3) is plotted for *Apis mellifera *(E) and *Danio rerio *(F).

**Figure 4:**
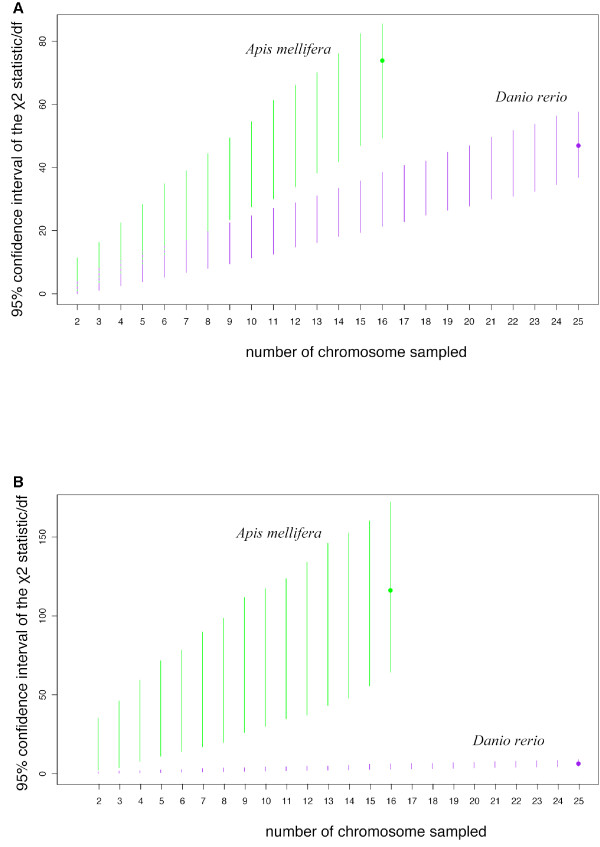
**χ^2 ^statistics of independence for the microsatellite sequences of the reference genome (A) and χ^2 ^statistics obtained by comparing 454 pyrosequencing data with the reference genome (B) for the chromosome × chromosome region two-way table**. The X-axis shows the number of bootstrapped chromosomes (2 to S, S being the chromosome number of the species considered), Y-axis: 95% confidence interval (based on 1,000 replicates) of the χ^2 ^statistic divided by degree of freedom. In green: *Apis mellifera*, in purple: *Danio rerio*.

Finally, we assessed the combined deviations of all four variables tested (to test for their association), by studying the distribution of the χ^2 ^statistic (representing the deviation from expected values). This approach made it possible to assess the deviations of 454 data distributions from the theoretical genome distribution for the two-, five-, and eight-probe enriched libraries (Figure 3). For example, for the chromosome × chromosome region interaction, χ^2 ^distributions did not differ significantly between the two-probe and five-probe enriched libraries, for either of the organisms considered (Kolmogorov-Smirnov test D = 0.088 P = 0.57 for *A. mellifera*; D = 0.052 P = 0.89 for *D. rerio*). However, the distribution obtained for eight-probe enrichment was significantly different from those for two- and five-probe enrichment, regardless of the genome considered (0.148 < D < 0.190; 0.005 < P < 0.008), although this difference became non-significant for an α-value = 0.01 before multiple testing (three pairwise tests, Figure 3). Moreover, for *D. rerio*, 75.6/73.6/61.2% of the χ^2 ^values (for the 2-, 5-, and 8-probe enriched libraries, respectively) was below the theoretical limiting value χ_1_^2 ^(3.84). This was not the case for *A. mellifera*, for which only 16.88/21.25/18.75% of the χ^2 ^values (for the 2-, 5-, and 8-probe enriched libraries, respectively) was below the 3.84 threshold. The homogenization involved in this procedure highlighted the significantly lower level of representativeness of *A. mellifera *libraries than of *D. rerio *libraries, regardless of the number of probes used. The difficulties experienced in correctly representing the *A. mellifera *genome resulted from the uneven distribution of microsatellites over the chromosomes and between different regions of the chromosomes, as shown by the large variance of the distribution of their probability of being found throughout the genome (Figure 3 and Figure 4). This bias was detected only for *A. mellifera *and may be further exacerbated by the sequence length distribution obtained during pyrosequencing, with an absence of fragments of more than 480 bp in length, as more than 53.8% of the complete genome sequences containing motifs in this species are of this size.

## Discussion

### Genome evolutionary dynamics and microsatellite representativeness

The composition and structure of genomes depend largely on the interaction of events in the evolution of a species and evolutionary processes, such as duplication [22,23,39], recombination and transposition [14]. The uniqueness of the resulting distribution of microsatellite loci makes direct comparison between model organisms very difficult and calls into question the generalization of the deviations in representativeness analyzed to even closely related taxa. We chose to analyze the representativeness of microsatellite distributions in the genomes of honey bee and zebrafish, not because these model organisms are emblematic and widely studied, but because they are the archetypes of genomes for which particular evolutionary events have had a major effect on the heterogeneity of genome composition and structure [24,40-42]. There may be many reasons for heterogeneous microsatellite contents, potentially leading to a loss of representativeness in the libraries isolated by methods such as that described here, combining several sensitive steps (enzyme digestion, multiplexing of the motif for enrichment, PCR, pyrosequencing). Despite the very large differences in both the proportions and occurrences of motifs on the chromosomes in the two reference genomes studied here (see Additional file 4, Figure S3), losses of representativeness in the enriched libraries obtained were found to be limited and of only marginal significance.

### Efficiency and representativeness of the method

Multiplexing with up to eight probes in enrichment phases greatly improves the isolation process in terms of the diversity of motifs obtained for a given cost or amount of time spent, and is of particular interest for evolutionary studies [43]. This phase is very flexible, with the possibility of using different panels of probes for different organisms of interest, thereby optimizing isolation according to current best knowledge about the organism and avoiding selection biases [33]. One potential concern is competition between probes during enrichment. Our results are reassuring in this respect, because no consistent bias related to the proportions of motifs was detected for either of the reference genomes. Indeed, motif proportions as high as 1/50 to 1/100 were correctly reproduced by this method, both in *Danio rerio *(e.g. 250/Mb AC *vs *2.5/Mb ACG) and in *Apis mellifera *(e.g. 250/Mb AG *vs *5/Mb ACG). This non biased distribution of the proportion of motifs between the sequenced libraries indicates that the efficiency and affinity of the multiplex probes used for enrichment were not density-dependent, as very large differences in motif proportions were correctly represented in the data obtained, even for very rare loci.

The heterogeneity of microsatellite sequences along chromosomes was largely reproduced in the data, although the global test highlighted a departure from the expected distribution. For example, chromosome 14, region 9 of *Apis mellifera *was found to have 11 times as many microsatellites as chromosome 16 region 1 in this species, but both regions were well represented in terms of proportions. More generally, we observed marginally significant departures from the reference distribution, and hence deviations in the representativeness of the data obtained empirically, whatever the heterogeneity in the distribution among regions of chromosomes and between chromosomes, for both model organisms. However, the intensity of the deviation, for most of the variables, was very limited given the very high level of heterogeneity of the reference genomes.

Moreover, the combination of motif proportions and heterogeneities in occurrence between chromosome regions were also well estimated. For example, the (AG) motif on chromosome 1 is 655 times more abundant than the ACAT motif on chromosome 12 for *A. mellifera *and the (AC) motif on chromosome 18 is 301 times more abundant than the (ACG) motif on chromosome 14 for *D. rerio*. In both cases the relative proportions of the corresponding loci were correctly assessed whatever the region and motif considered.

Finally, it could be argued that this method fails to deal with the distribution of isolated loci with fragment sizes greater than 480 bp. This is perfectly true, as we are constrained by advances in pyrosequencing technology, which is currently unable to yield longer sequences. At first glance, this might appear to be an important issue, but this is actually not the case when practical aspects are considered. Indeed, the size range currently provided by pyrosequencing (0 - 480 bp) encompasses the sizes of fragment currently used to design microsatellite markers for analysis anyway.

Overall representativeness is not a trivial outcome, as multiple artifacts may bias the overall result during the process, making it impossible to decipher the causes of distortions. Probe enrichment efficiency did not depend on the density of sequences with target motifs. This and the small number of PCR cycles used ensured that no technical issue resulted in biased estimates of microsatellite genome content. The multiplexing strategy of motif enrichment followed by 454 pyrosequencing used here therefore appears to be a very promising technique for improving the development of microsatellite markers.

## Conclusions

The possibility of unraveling biases related to molecular techniques in the different organisms remains a rare opportunity, even for the geneticists of today. This study of the contrasting model organisms honeybee and zebrafish revealed common misrepresentations of sequence length distributions due to 454 Titanium series pyrosequencing. There may be a number of reasons for this lack of representativeness, including enzymatic digestion, enrichment, or emulsion-PCR differential efficiencies. Sequence lengths may also be shortened by the microsatellites themselves, as the sequencing process cycles nucleotides one after another. Progress and hypothesis-testing, based on the conclusions drawn here, will undoubtedly lead to rapid improvements in the process. However, as the detected potential sequence length biases do not make it impossible to obtain usable microsatellite loci, this drawback does not invalidate the process as a whole. The simultaneous analysis of two organisms (*A. mellifera *and *D. rerio*) with profoundly different genome features provided important information about the method used. Indeed, we found that although genome features may be misrepresented, particularly for *A. mellifera*, which displays a high level of heterogeneity, the general trends of distributions are conserved in the experimental acquisition of sequences, even when the genomes differ in as extreme a manner as those studied here. Even more important for most potential projects, the good fit to reference data obtained for over proportions of motifs, in both organisms, and the very large number of loci obtained, are encouraging. All these features should ensure a promising future for this method in the high-throughput isolation of microsatellites.

## Methods

### Multiplex-enriched library preparation

Microsatellites were isolated with a modified version of a widely used biotin-enrichment protocol [29] at GenoScreen (Lille). Genomic DNA was extracted from 25 mg of tissue with the DNeasy Tissue Kit (QIAGEN) and treated with RNase. The genomic DNA (2 μg) obtained was digested with *Rsa*I (Fermentas) for 1 hour at 37°C, according to the manufacturer's instructions. Standard adapters were then ligated to the fragmented DNA and samples were purified on a Nucleofast PCR plate (Macherey-Nagel). Depending on the library prepared, biotin-labeled oligonucleotides corresponding to a mixture of 2, 5 or 8 microsatellite motifs were hybridized to the ligated DNA for 10 min after initial denaturation of the ligation (motifs for 2-probe enrichment were: AC and AG; motifs for 5-probe enrichment were: AC, AG, AAC, AGG, ACAT; and motifs for 8-probe enrichment were: AC, AG, AAC, AGG, ACAT, AAG, ACG and AGAT). The enrichment step was carried out with Dynabeads (Invitrogen). The resulting enriched DNA was amplified with standard adapters, over 25 cycles (20 s at 95°C, 20 s at 60°C and 90 s at 72°C), with a final elongation step of 30 min at 72°C. The polymerase chain reaction (PCR) product was immediately purified with the QIAquick PCR purification kit (QIAGEN).

### 454 library Titanium sequencing

In total, 1 μg of each purified enriched library was used for 454 FLX Titanium libraries (Roche Applied Science) preparation, according to the manufacturer's protocols, at Genoscreen (Lille, France). Emulsion PCR (emPCR) was carried out at a ratio of 1 copy per bead, with subsequent disruption with isopropanol. Beads containing amplified DNA fragments were enriched and recovered for sequencing, to provide 50,000 to 70,000 enriched beads for each library. The recovered ssDNA beads were packed onto region 1/16 of a 70 mm × 75 mm Titanium PicoTiter plate and sequenced with 200 cycles. Sample preparation and analytical processing, such as base calling, were performed at Genoscreen (Lille, France), according to the manufacturer's protocol for the Titanium series.

### Bioinformatics 454 pipeline to primer design

We used QDD software [37] to select the 454 sequences for primer design. Enrichment adaptors were removed from sequences, and sequences longer than 80 bp and containing at least four repeats of perfect microsatellites, for any 2 to 6 bp motif, were selected for further analysis. Sequence similarities were identified by an "all against all" BLAST [44,45] analysis, using an e-value of 1E-40 and with microsatellite sequences soft-masked. Sequences for which pairwise similarity in the flanking regions exceeded 95% were grouped into contigs and a 2/3 majority rule consensus sequence was created from each contig. Sequences with significant BLAST hits to other sequences and an overall similarity in the flanking region of less than 95% were discarded to avoid potential intragenomic multicopy sequences. All unique sequences (with no BLAST hit to any other 454 read) and consensus sequences were screened for the presence of short repetitions in the flanking regions. PCR primers were designed only if the target microsatellite had at least five repeats, the PCR product was between 90 and 320 bp in length and the flanking region contained, at most, one four-base mononucleotide stretch or two repeats of any di-hexa base-pair motif.

### Statistical analysis -- representativeness of 454 data

Two datasets were constructed for each of the organisms studied (*A. mellifera *and *D. rerio*). For each species, the first dataset corresponded to the reference genome: the complete published genome digested *in silico *with *Rsa*I. For the eight motifs chosen for this analysis, all perfect microsatellites with at least four repeats were identified and mapped in the genomes of *A. mellifera *and *D. rerio*. The second data set corresponded to all 454 reads containing microsatellites subjected to Blast analysis against the corresponding genome (MEGABLAST, e-value 1E-40). All 454 reads with a high quality hit against the genome (at least 90% of the length of the 454 sequence was aligned by MEGABLAST) were mapped within the genome and their distribution established as for the complete genome. Each of the two datasets consisted of a four-way contingency table [n_ijkl_]_ijkl_, where n_ijkl _is the number of microsatellites corresponding to each variable describing genome features, and *i *refers to the chromosome and varies from 1 to the total number of chromosomes in the species considered, n = 16 for *Apis mellifera *and n = 25 for *Danio rerio*; *j *is the chromosome region (chromosomes were divided into 10 regions of equal length); *k *is the length of sequences containing repeated motifs. More precisely, fragment lengths were classified into five groups: 80 (81 to 160 bp), 160 (161 to 240 bp), 240 (241 to 320 bp), 320 (321 to 400 bp), and 400 (401 to 480 bp). We removed the 0-80 range, due to the uncertainties involved in Blast analyses of shorter fragments against genomes, and the > 480 range, due to the small number of 454 reads of that length. Finally, *l *is the proportion of microsatellite motifs for the eight selected motifs: AC, AG, AAC, AAG, ACG, AGG, ACAT, and AGAT.

The microsatellite distribution obtained with 454 data was compared with that of the published genome, used as the reference distribution:

$${\hat{p}}_{ijkl}=\frac{n_{ijkl}}{n}=f_{ijkl}$$

where *p*_*ijkl *_is the reference genome probability of a microsatellite being found for a particular combination of variables *i*, *j*, *k *and *l*; *n_ijkl _*is the number of microsatellites found in the reference genome for a particular combination of variables *i*,*j*,*k *and *l*; is the total number of microsatellites and *f_ijkl _*is the observed frequency of microsatellites found for a particular combination of variables *i*, *j*, *k *and *l*. Comparisons were carried out by χ^2 ^tests for both one-way and two-way tables extracted from the four-way dataset. As general tendencies were of particular interest in this analysis, we focused here on the one-way and two-way tables. When necessary, permutation tests were used to approximate the distribution of the χ^2 ^statistic. When the global test result was significant, multiple tests on each cell were performed. We controlled for false positives, using the false discovery rate (FDR), with Benjamini and Hochberg correction [46].

Furthermore, the potential effect of the number of chromosomes on genome heterogeneity was assessed for both organisms, with the use of a bootstrap resampling procedure to estimate the test statistics corresponding to the contingency table "Chromosome × chromosome region". For each genome (*A. mellifera *and *D. rerio*), we plotted the 95% confidence interval of the χ^2 ^statistic for tests (see Additional file 1) between the reference distribution, based on the independence of the variables, and the observed genome classes, and between the observed genome values and the observed 454 data. In each genome, we selected *r *= 2 to S chromosomes (where S is the total number of chromosome) and sampled, 1,000 times, *r *chromosomes within S, using the bootstrap resampling method [47]. For each value of *r*, we therefore obtained a bootstrapped distribution of the χ^2 ^statistic.

Finally, the combination of observed deviations for all four variables tested was evaluated, by studying distributions of the χ^2 ^statistic (representing deviations from expected values). This made it possible to assess the deviations of 454 data distributions from the reference genome distribution for the two-, five-, and eight-probe enriched libraries. For both organisms, the distributions of the statistics were investigated with the Kolmogorov-Smirnov test [48].

## Authors' contributions

JFM conceived the study and wrote the manuscript. NP participated in the design of the study and performed the statistical analysis. EM participated in the design of the study and performed the bioinformatics analysis. SF participated in the design and performed the molecular biology. CC participated in the design and helped to write the manuscript. VD performed molecular biology experiments and helped to write the manuscript. TM conceived the study and helped to write the manuscript. AG conceived the study and helped to write the manuscript (contribution equal to that of the first author). All authors have read and approved the final manuscript.

## Supplementary Material

Additional file 1 **Additional information and methods**. this file contains additional information about genomic distribution of microsatellites and its modeling. Click here for file

Additional file 2 **Representativeness of genome distribution after digestion with *Rsa*I**. Representativeness of genome distribution after digestion with *Rsa*I in the case of *Apis mellifera *(A) and *Danio rerio *(B) for each of the six considered two-way tables: chromosome × chromosomic region, chromosome × length of sequences, chromosome × microsatellite motifs, chromosomic region × length of sequences, chromosomic region × microsatellite motifs and length of sequences × microsatellite motifs. "Stat" refers to the overall χ^2 ^statistic for the test of independence between the two variables defining the table. For each cell of the table: a red dot corresponds to an observed number significantly higher than expected under the hypothesis of independence between the two variables defining the table, a blue dot corresponds to an observed number significantly lower than expected under the hypothesis of independence between the two variables defining the table; absence of dot means a non-significant deviation. The size of the dot is inversely-proportional to the P-value. The scaling is computed for the six considered tables so comparison of significance between tables is meaningful. For cell tests, Benjamini-Hochberg^20 ^correction for multiple tests was performed. Click here for file

Additional file 3 **Comparison of genome features using the equiprobability model **Comparison of genome features using the equiprobability model in the case of *Apis mellifera *(A) and *Danio rerio *(B) for each of the four considered one-way tables: chromosome, chromosomic region, length of sequences and microsatellite motifs. For each table is figured in red the 95% confidence interval of the expected number of microsatellites under the equiprobability hypothesis, in black are figured observed numbers (genome distribution after digestion *in silico *with *Rsa*I). Click here for file

Additional file 4 **Representativeness of genome features using the equiprobability model**. Representativeness of genome features using the equiprobability model in the case of *Apis mellifera *(A) and *Danio rerio *(B) for each of the six considered two-way tables chromosome × chromosomic region, chromosome × length of sequences, chromosome × microsatellite motifs, chromosomic region × length of sequences, chromosomic region × microsatellite motifs and length of sequences × microsatellite motifs. "Stat" refers to the overall χ^2 ^statistic for the test of independence between the two variables defining the table. For each cell of the table: a red dot corresponds to an observed number significantly higher than expected under the hypothesis of independence between the two variables defining the table; a blue dot corresponds to an observed number significantly lower than expected under the hypothesis of independence between the two variables defining the table; absence of circle means a non-significant deviation. The size of the dot is inversely-proportional to the P-value, the scaling being made to the six considered tables so that the comparison of sizes between tables is meaningful. For cell tests, Benjamini-Hochberg^20 ^correction for multiple tests was performed. Click here for file

Additional file 5 **454 reads for *A. mellifera *and *D. rerio ***. this file contains the raw 454 FASTA reads in both model species for 2-, 5- and 8-probe enrichment sequences. Click here for file
