# Supplementary material for: Representativeness of microsatellite distributions in genomes, as revealed by 454 GS-FLX Titanium pyrosequencing
Source: BMC Genomics. 2010 Oct 12;11:560. doi: 10.1186/1471-2164-11-560 (PMC3091709; doi:10.1186/1471-2164-11-560)
Supplement: Additional file 1 — Additional information and methods. this file contains additional information about genomic distribution of microsatellites and its modeling. [file 1471-2164-11-560-S1.DOCX]

## Motif selection for probe design

Thirteen published sequenced genomes or Whole Genome Shotgun sequences among the organisms of interest for the team were screened for microsatellite abundance (insects: *Apis mellifera, Anopheles gambiae, Drosophila melanogaster, D. yakuba, D. simulans, Bombyx mori, Tribolium castaneum*; Vertebrates: *Takifugu rubripes, Danio rerio, Gallus gallus, Bos taurus, Mus musculus, Rattus norvegicus*). Motifs that were among the 12 most frequent motifs for each genome were identified and classified in decreasing order (Table S1). From this pool of motifs eight were selected and the following eight probes were designed for enriching *A. mellifera* and *D. rerio* total DNA extractions : (AAC)7A, (TG)10, (TC)13, (AAG)8A, (AGG)6, (ACG)5AC, (ACAT)9, and (ATCT)10AT. This selection was based on melting temperature compatibility (between 56 and 59°C), and avoiding motifs that were likely to produce hairpin structures even among the most frequent ones (e.g. AT, CG, and AAT). In the multiplex enriched libraries, motifs for 2-probes were: AG and AC; motifs for 5-probes libraries were: AG, AC, AAC, AGG, ACAT; and motifs for 8-probes libraries were: AG, AC, AAC, AGG, ACAT, ACG, AAG and ATCT.

## Table S1: Proportions of microsatellite motifs in selected genomes

Occurrence of the twelve most frequent motifs for each of the 13 complete genomes or Whole Genome Shotgun sequences. Complete genomes: *Apis mellifera* (Am)*, Anopheles gambiae* (Ag)*, Drosophila melanogaster* (Dr)*, D. yakuba* (Dy)*, D. simulans* (Ds)*, Tribolium castaneum* (Tc), *Danio rerio* (Dr)*, Gallus gallus* (Gg)*, Bos taurus* (Bt)*, Mus musculus* (Mm)*, Rattus norvegicus* (Rn); Whole Genome Shotgun sequences: *Takifugu rubripes* (Tr)*, Bombyx mori* (Bm). We selected *Apis mellifera* and *Danio rerio* respectively as invertebrate/vertebrate model organisms for the experimental/ theoretical comparisons.

| **MOTIF** | **Am** | **Ag** | **Dm** | **Ds** | **Dy** | **Bm** | **Tc** | **Tr** | **Dr** | **Gg** | **Mm** | **Rn** | **Bt** | **rank** | **score** |
| --- | --- | --- | --- | --- | --- | --- | --- | --- | --- | --- | --- | --- | --- | --- | --- |
| 1 | AT | AC | AC | AC | AC | AT | AAT | AC | AC | AT | AC | AC | AC | AC | 13 |
| 2 | AG | AG | AT | AT | AT | AC | AC | AG | AT | AC | AG | AG | AT | AT | 13 |
| 3 | AAT | ACG | AG | AG | AG | AG | AT | AT | AAT | AG | AT | AT | AG | AG | 13 |
| 4 | AC | AGC | AGC | AGC | ACG | AAT | AG | AGG | AG | AAT | AAAC | AGAT | AGC | AAT | 13 |
| 5 | AAG | AAG | ACG | ACG | AGC | ACT | AAAT | AAT | AGAT | AAC | AAAG | AAC | ACG | AAC | 11 |
| 6 | AGG | CG | AAC | AAC | AAC | AAAT | CCG | AGAGG | AAAT | AAAC | AAC | AGG | AAC | AGG | 8 |
| 7 | CG | AT | AAT | AAT | AAT | AGT | AAG | AGGT | ACAG | AAAT | AGG | AAAC | AAT | AGC | 8 |
| 8 | AAAG | AAT | AGT | AGT | ACC | AAG | AAC | ACCT | AATAT | AAAG | AAGG | AAAG | AACTG | AAG | 8 |
| 9 | AAAT | ACC | ACT | AGG | AGG | CG | AATT | AGC | ACCT | AAGG | AGAT | AAAT | AAGTC | AAAT | 8 |
| 10 | AAGG | AAC | ACC | ACT | AGT | AAAC | CG | ACG | AGGT | AGG | AAAT | AAGG | ACC | ACG | 7 |
| 11 | ACG | AGT | AGG | ACC | ACT | AAC | AGC | ACAG | AAC | AAG | AAG | ACC | AAAT | ACC | 7 |
| 12 | AGC | ACT | AAG | AAG | CCG | ACAT | ACC | AGT | AATG | AAAAC | AAT | AAT | AAAC | AGT | 6 |

## Microsatellite distribution across *Rsa*I digested genomes

The use of a restriction enzyme is common to most DNA library preparations, including microsatellites isolation. Although this technique is of great interest in preparing total genomic DNA, what confidence do we have about the representativeness of the genome accessible in libraries built only from fragments obtained after *Rsa*I digestion? This question was addressed by analyzing the microsatellite distribution across genomes. We compared the published genome of *A. mellifera* and *D. rerio* digested by *Rsa*I *in silico* to theoretical distributions.

The comparisons were performed by the way of χ² tests for both one-way and two-way tables extracted from the four-way data set (see Methods for definitions). If necessary, permutation tests were used in order to estimate the distribution of the χ² statistic. When global test was significant, multiple tests from each cell were performed. To control for false positives, we used the false discovery rate (FDR) and its modification as proposed by Benjamini and Hochberg (1995).

For one way tables, we used uniform theoretical distributions in the *Rsa*I *vs* equiprobability framework and observed probabilities distributions in testing independence of variables. For two way tables two theoretical distributions were considered. First, if p_ij_ refers to the probability of a cell in a considered contingency table, we call independence hypothesis: (H_0_): p_ij_ = p_i._ x p_.j_ where p_i._ is the marginal probability estimated by

$$\frac{\sum_{j} n_{ij}}{n}$$

where $n$ is the total number of microsatellites.

### Independence of the variables for *in silico* *Rsa*I digested genomes

We tested the independence of the four variables in each genome digested by the *Rsa*I restriction enzyme. The probabilities for each variable are estimated using the data obtained by *in silico* *Rsa*I digestion of the *A. mellifera* and *D. rerio* genomes.

We first defined whether the potential differences between the two model organisms could be due to differences in chromosome number (genome size and information heterogeneity). First, the number of microsatellites in both genomes was highly correlated with the length of the chromosomes (r=0.97, P< 2.2 e-16 for *D. rerio* and r=0.95, P< 1.9 e-08 for *A. mellifera*). Second, the heterogeneous distribution of the microsatellites number on the chromosomic position differed according to the organism considered (Additional file 2, Figure S1). Any chromosome content in *D. rerio* is a good estimator of the rest of the genome (the 24 other chromosomes) when for *A. mellifera* each chromosome brings its own information and variability due to the number of microsatellites in stochastic hot spots (Additional file 2, Figure S1).

All the variables were mutually linked (Additional file 2, Figure S1). The associations for microsatellites abundance between the variables chromosome and chromosomic position was highly significant whatever the genome considered (respectively stat = 11 727.96, P<10^-5^; stat = 11 813.3, P<10^-5^). Microsatellites loci, whatever the interactions of variables considered, were not randomly distributed along the genome and hence prevented from direct comparisons between the two organisms as is allowed by the equiprobability model.

### Representativeness of *Rsa*I digested genome *vs* equiprobability model

Since the *Rsa*I restriction site (GT^AC) does not correspond to any of the eight motifs for which the genomes were enriched, the digestion with this enzyme does not interfere with the isolation process. However, we compared the two organisms for their genome content in microsatellites (after digestion with *Rsa*I restriction enzyme) with a uniform theoretical distribution of microsatellites along the genome considering four variables assumed to be mutually independent:

*i* refers to chromosome and varies from 1 to the total chromosome number of the considered species, n=16 for *Apis mellifera* and n=25 for *Danio rerio*, where Pm_c_ is the probability for a microsatellite to be found on a given chromosome is proportional to the length of the considered chromosome. (p’_i_ = chromosome size/ genome size);

*j* is the chromosomic region (chromosomes are divided into 10 regions of equal lengths) and P_j_ is the probability for a microsatellite to be found on a given chromosomic region (each chromosome is divided in 10 regions therefore p’_j_ = 0.1);

*k* is the length of sequences that contained repeated motifs. More precisely, fragments lengths were defined using five modalities: 0 (1 to 80 bp), 80 (81 to 160 bp), 160 (161 to 240 bp), 240 (241 to 320 bp), 320 (321 to 400 bp), 400 (401 to 480 bp), 480 (>481) therefore p’_k_ = 0.143. We recognize that the >480 bp range potentially contains much more information than the other ranges but, as the sequences lengths for Titanium series is currently limited to 480 bp, we consider >480 bp range as “not usable range” and hence do not give it any particular weighting;

*l* is the microsatellite motifs proportion of the eight selected motifs: AC, AG, AAC, AAG, ACG, AGG, ACAT, AGAT where P_l_ is the probability for a motif to be found, therefore p’_l_ = 0.125.

We call equiprobability distribution hypothesis (H_0_): p_ij_ = p’_i_ x p’_j_ where p’_i_ (resp. p’_j_) refers to the equiprobability distribution, except for chromosome length where we weighted with the length of each chromosome. In this way, we can test the equiprobability distribution hypothesis for each pair of variables.

With these assumptions in mind, rejecting H_0_ implies a deviation from the equiprobability.

It is worth noting that *A. mellifera* and *D. rerio* are not structured in the same way. Indeed, *A. mellifera* genome displays one over-represented motif (AG motif) and the *D. rerio* present two over-represented motifs (i.e. AC and AG motifs) as shown in Additional file 3, Figure S2. Considering sequence lengths (obtained by *Rsa*I restriction enzyme), both *A. mellifera* and *D. rerio* display an over-represented number of microsatellites in the range >480 (whatever the chromosome considered) as shown in Additional file 3, Figure S2. However, in *D. rerio*, the ranges 80-160 and 161-240 also display an over-represented number of microsatellites. In the same time, for this particular genome 27.5 % of the microsatellites are in the range >480 when it represents 53.8% for *A. mellifera* (Additional file 3, Figure S2). Furthermore, chromosomes of *A. mellifera* display numerous stochastic high densities in microsatellite as well as an unbalanced distribution of loci in one arm for most chromosomes (Additional file 4, Figure S3). Contrastingly, the *D. rerio* genome as cut by *Rsa*I over-represents microsatellite abundance in the two extreme parts of the chromosomes (range 1 and 9-10) that correspond to the telomere regions of each arm (Additional file 4, Figure S3).

## Application of the High throughput microsatellites isolation to *Zingel asper* (Linnaeus, 1758) [Actinopterygii: Perciformes: Percidae]

As a validation of the methodology presented in this paper, we used the same procedure as detailed in the Methods section to isolate *de novo* microsatellites in a highly protected fish species *Zingel asper*. The following results are detailed in Table S2. Among the 15 004 sequences generated by the GS-FLX 454 Titanium run for this species, 272 sequences were retained by QDD (available on request, submitted) using the following criteria: perfect motifs, number of repeat ≥ five, short repetition free flanking regions ≥ 30 base pairs. Among the selected sequences, we designed 241 primer pairs of microsatellites for which amplicons presented a length ≥ 100 base pairs. Amplifications were performed for 181 primers pairs in a total volume of 10µL containing 2µL of 1/10^th^ diluted total DNA extract (obtained from Puregen Gentra Tissue Kit, QIAGEN) using QIAGEN Multiplex PCR Kit following the manufacturer's protocol. Thermocycling was done on a Mastercycler^®^ (Eppendorf) with the following protocol: 95°C for 10min, followed by 30 cycles (94°C for 1min, 56°C for 1min, 72°C for 1min), and 60°C for 45min. Based on agarose gel migration, 105 primer pairs associated with clear amplification pattern were retained. All 105 loci were hereafter amplified separately using forward primers labeled with fluorescent dyes 6-FAM (Eurogentec), PET, NED or VIC (Applied Biosystems). Visualization of the amplicons was conducted on an ABI 3130 Genetic Analyzer (Applied Biosystems).

Alleles sizes were scored against an internal GeneScan-500 LIZ^®^ Size Standard and genotypes were obtained using GeneMapper^®^ 3.7 (Applied Biosystems). Finally, only loci displaying a number of alleles ≥ 2 among 8 individuals where retained. Following this analytical step, a total of 60 polymorphic loci were selected. The use of the methods described in this paper therefore produce efficient and rapid working primers for microsatellite loci in a species for which the genome is unknown.

## Table S2: summarized results for the microsatellites isolation in *Zingel asper*

| **454 input sequences** | **Sequences retained by QDD^1^** | **Sequences for which primers were designed^2^** |
| --- | --- | --- |
| 15 004 | 272 | 241 |
| **PCR tested loci^3^** | **Loci associated with clear amplification pattern^4^** | **Nb of loci retained^5^** |
| 181 | 105 | 60 |

^1^ Criteria: perfect motifs; ≥ 5 repeats; ≥ 30 bp for flanking regions without short repetitions.

^2^ Criteria: ≥ 100 bp length for amplicons

^3^ 60 loci with 5 or 6 repeats were discarded.

^4^ Based on agarose gel migration

^5^ Criteria: ≥ 2 alleles per locus among 8 individuals; unambiguous genotype profile after analysis of fluorescent Dye labeled amplicons on an ABI 3130 Genetic Analyzer (Applied Biosystems) and GeneMapper 3.7 (Applied Biosystems).
