# Supplementary figures and images for: Representativeness of microsatellite distributions in genomes, as revealed by 454 GS-FLX Titanium pyrosequencing
Source: BMC Genomics. 2010 Oct 12;11:560. doi: 10.1186/1471-2164-11-560 (PMC3091709; doi:10.1186/1471-2164-11-560)

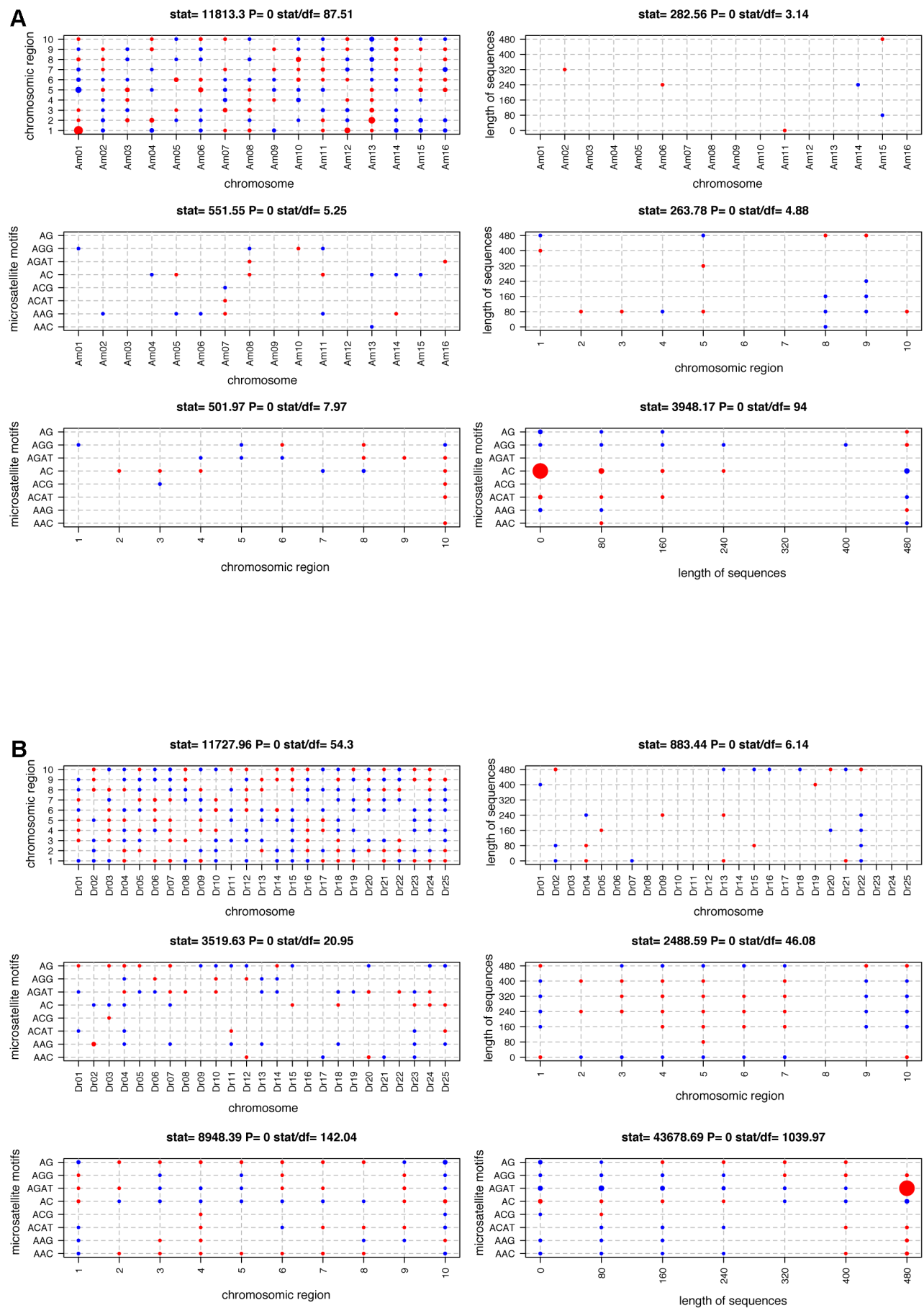

Supplement: Additional file 2 — Representativeness of genome distribution after digestion with RsaI. Representativeness of genome distribution after digestion with RsaI in the case of Apis mellifera (A) and Danio rerio (B) for each of the six considered two-way tables: chromosome × chromosomic region, chromosome × length of sequences, chromosome × microsatellite motifs, chromosomic region × length of sequences, chromosomic region × microsatellite motifs and length of sequences × microsatellite motifs. "Stat" refers to the overall χ2 statistic for the test of independence between the two variables defining the table. For each cell of the table: a red dot corresponds to an observed number significantly higher than expected under the hypothesis of independence between the two variables defining the table, a blue dot corresponds to an observed number significantly lower than expected under the hypothesis of independence between the two variables defining the table; absence of dot means a non-significant deviation. The size of the dot is inversely-proportional to the P-value. The scaling is computed for the six considered tables so comparison of significance between tables is meaningful. For cell tests, Benjamini-Hochberg20 correction for multiple tests was performed. [file 1471-2164-11-560-S2.PDF]

**A**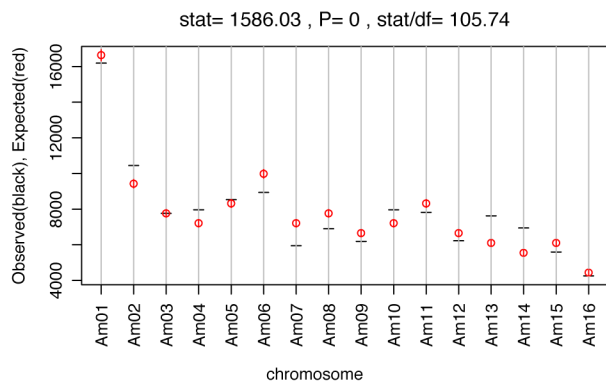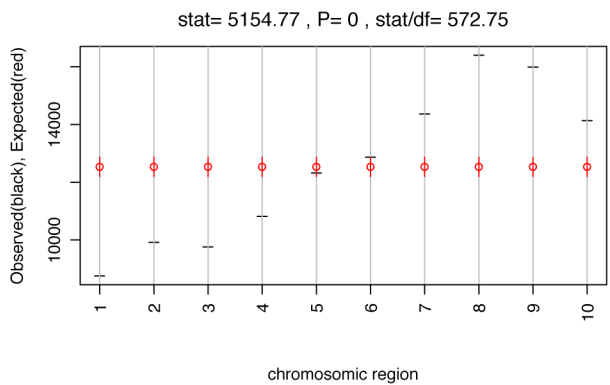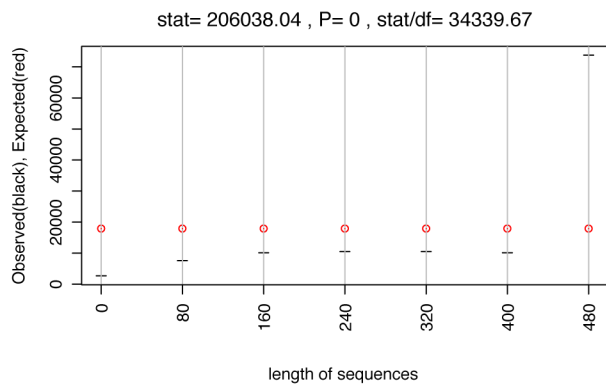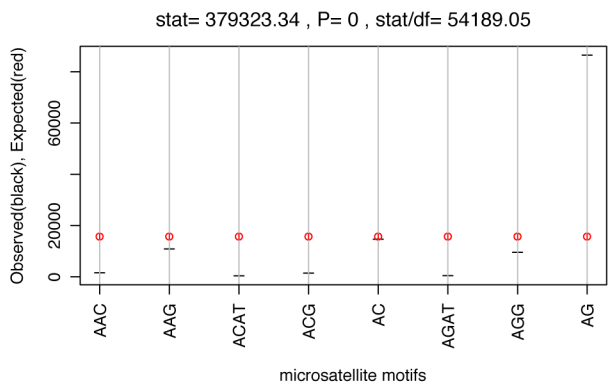**B**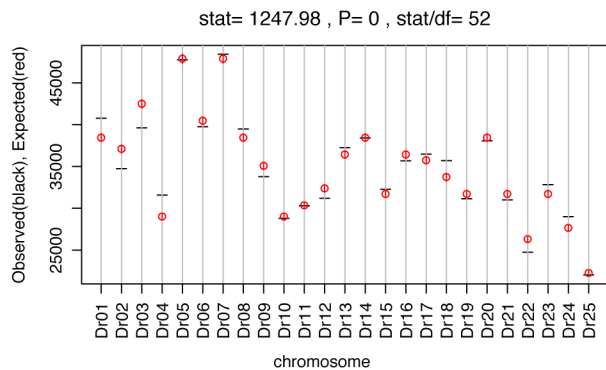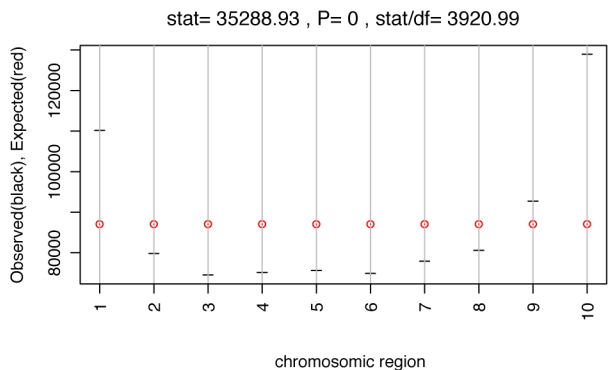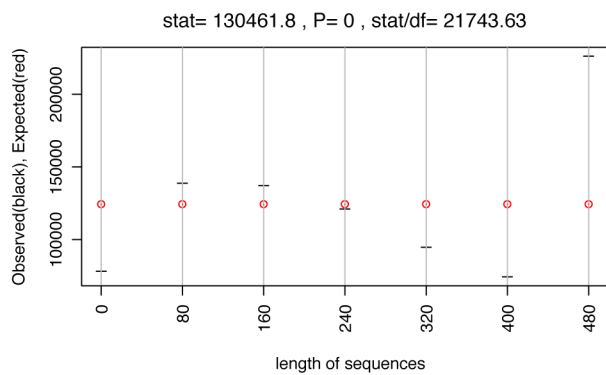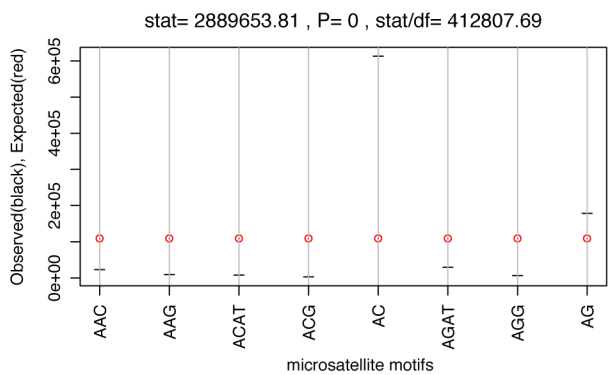

Supplement: Additional file 3 — Comparison of genome features using the equiprobability model Comparison of genome features using the equiprobability model in the case of Apis mellifera (A) and Danio rerio (B) for each of the four considered one-way tables: chromosome, chromosomic region, length of sequences and microsatellite motifs. For each table is figured in red the 95% confidence interval of the expected number of microsatellites under the equiprobability hypothesis, in black are figured observed numbers (genome distribution after digestion in silico with RsaI). [file 1471-2164-11-560-S3.PDF]

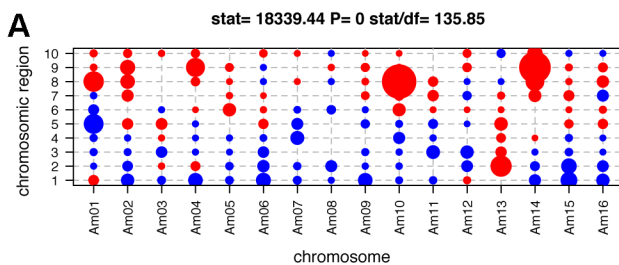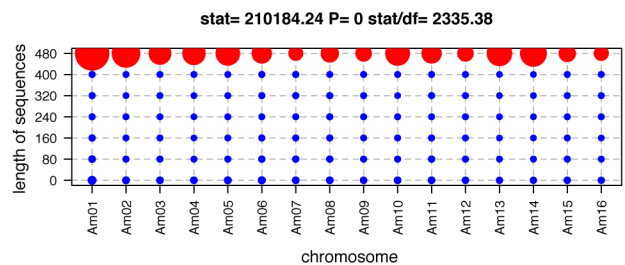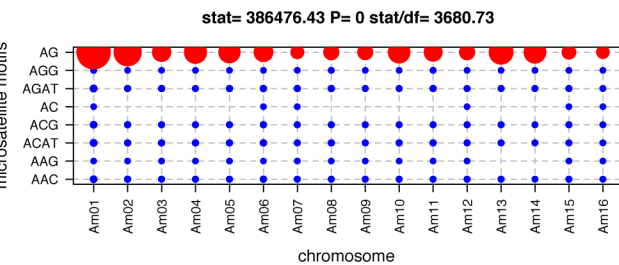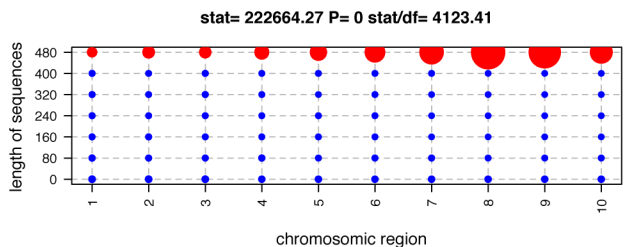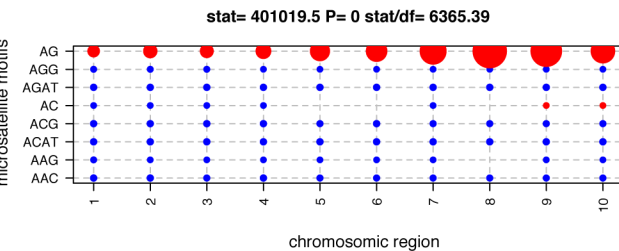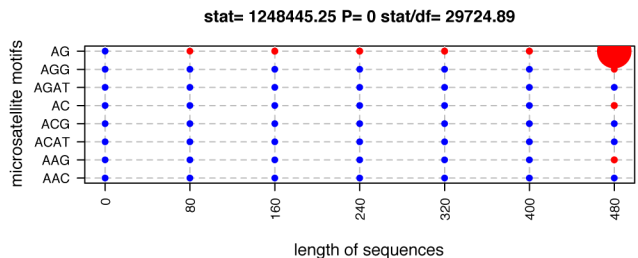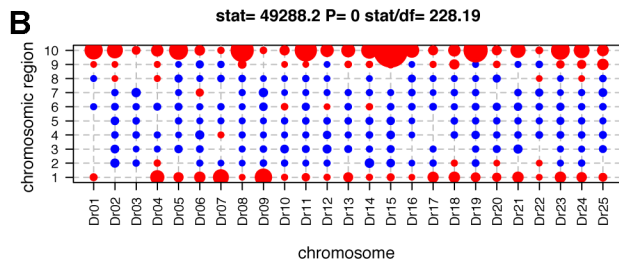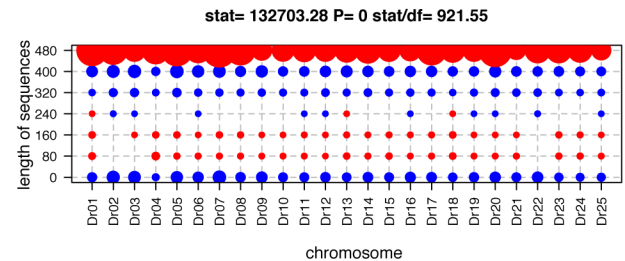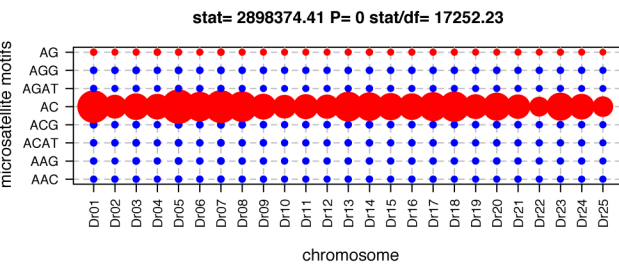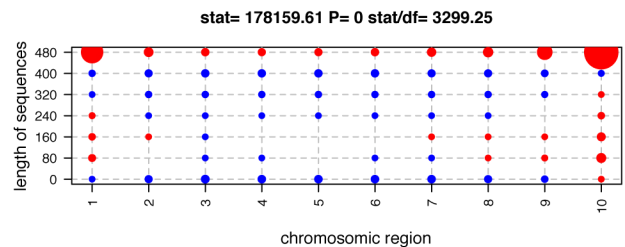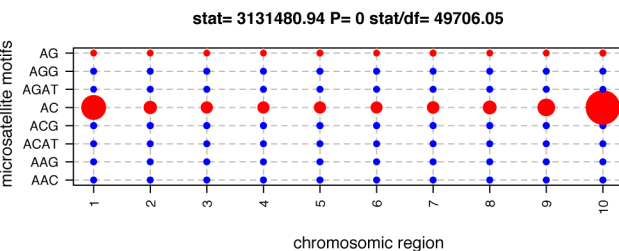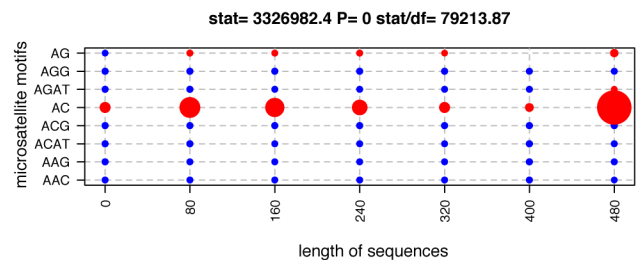

Supplement: Additional file 4 — Representativeness of genome features using the equiprobability model. Representativeness of genome features using the equiprobability model in the case of Apis mellifera (A) and Danio rerio (B) for each of the six considered two-way tables chromosome × chromosomic region, chromosome × length of sequences, chromosome × microsatellite motifs, chromosomic region × length of sequences, chromosomic region × microsatellite motifs and length of sequences × microsatellite motifs. "Stat" refers to the overall χ2 statistic for the test of independence between the two variables defining the table. For each cell of the table: a red dot corresponds to an observed number significantly higher than expected under the hypothesis of independence between the two variables defining the table; a blue dot corresponds to an observed number significantly lower than expected under the hypothesis of independence between the two variables defining the table; absence of circle means a non-significant deviation. The size of the dot is inversely-proportional to the P-value, the scaling being made to the six considered tables so that the comparison of sizes between tables is meaningful. For cell tests, Benjamini-Hochberg20 correction for multiple tests was performed. [file 1471-2164-11-560-S4.PDF]
